# Dr. Charles W. Kingsley

**Published:** 1893-12

**Authors:** R. Ottolengui


### Obituary.

### DR. CHARLES W. KINGSLEY.

All the profession will learn with regret that Dr. Charles W. Kingsley died in Paris on the 22d of October. He was ill less than two weeks suffering from pneumonia and congestion of the lungs. The crisis was supposed to have been passed in safety, when heart-failure suddenly supervened.

Dr. Kingsley was born December 3, 1841, at Pittsford, Vermont. While preparing himself for college he earned the necessary fees by teaching, and, finally, while a student of the University of Rochester the Civil War began, and he enlisted in the Union Army in 1864, remaining until he was mustered out in 1865. In the same year he began the study of dentistry in the office of his brother, Dr. Norman W. Kingsley, and because of a natural aptitude rapidly became very skilful both as an operator and in other branches of dental work. He was the assistant and associate of his brother for five years, and in December, 1871, formed a partnership with Dr. Crane, in Paris. This partnership continued until it expired by limitation, thirteen years later, since which time he was in independent practice at No. 9 Rue Auber.

During his career in Europe Dr. Charles Kingsley acquired the distinction of being considered one of the most skilful Americans practising abroad, and rapidly obtained a very large and remunerative practice, his clientèle being of the most select order, including many of the nobility from various capitals of Europe, and the most distinguished Americans travelling abroad.

Among his brother dentists he was held in high esteem, and contributed as greatly as any towards maintaining the character, dignity, and good name of American dentists in Europe. He was vice-president of the American Dental Society in Paris, of which Dr. Thomas W. Evans is president.

He was a man of studious habits, refinement of manner and culture, a master of his profession, and of sterling integrity.

He married Miss Jessie Bradbrook in 1871, and she survives him. He also leaves two sons, the elder of which is at Yale University, and the younger at school in England. His body was cremated at Père la Chaise October 24.

R. Ottolengui.

